# Self-Assembled PLGA-Pluronic F127 Microsphere for Sustained Drug Release for Osteoarthritis

**DOI:** 10.3390/ph17040471

**Published:** 2024-04-07

**Authors:** Semee Seon, Yixian Li, Sangah Lee, Yoon Sang Jeon, Dong Seok Kang, Dong Jin Ryu

**Affiliations:** 1 Research & Development, Nextbiomedical Co., Ltd., Incheon 22013, Republic of Korea; 2 Orthopedic Surgery, Inha University Hospital, Incheon 22013, Republic of Korea

**Keywords:** PLGA drug, sustained-release drug, osteoarthritis, local injection

## Abstract

For many years, sustained-release drug delivery systems (SRDDS) have emerged as a featured topic in the pharmaceutical field. Particularly for chronic diseases, such as osteoarthritis, there is a lot of demand for SRDDS because of the long treatment period and repetitive medication administration. Thus, we developed an injectable PLGA-F127 microsphere (MS) that is capable of the in situ conversion to an implant. The microprecipitation method for PLGA-F127 MS was established, and the physicochemical stability of the products was confirmed. The microspheres were assembled into a single mass in 37 °C aqueous conditions and showed a remarkably delayed drug release profile. First, the release started with no significant initial burst and lagged for 60 days. After that, in the next 40 days, the remaining 75% of the drugs were constantly released until day 105. We expect that our PLGA-F127 MS could be employed to extend the release period of 2 months of medication to 4 months. This could be a valuable solution for developing novel SRDDS for local injections.

## 1. Introduction

For osteoarthritis (OA), as a low-grade chronic inflammatory disease, and rheumatoid arthritis (RA), as a chronic autoimmune inflammatory disease, intraarticular injection of corticosteroid is still one of the most effective options for pain control and decreasing inflammatory reactions [1,2,3]. High doses of corticosteroid induce a catabolic reaction in cartilage, and repeated injections could cause septic arthritis [4,5,6]. Therefore, developing sustained-release injection materials that can gradually release low-dose steroids and be maintained over a long duration are necessary [7]. Meanwhile, the development of sustained-release injections using various materials has been attempted, and triamcinolone acetonide (TA) sustained-release injections using Polylactic-co-glycolic acid (PLGA) microspheres have actually been developed (Zilretta, Flexion Therapeutics, Burlington, MA, USA) [8,9]. It is known to act in the joint space for about three months [10]. However, the TA showed stronger cytotoxic effects on chondrocytes and tenocytes than dexamethasone (DXA) in high doses [11,12]. When developing a sustained-released DXA injection, it can be expected to be used as an effective arthritis treatment while reducing the side effects of TA.

Sustained-release drug delivery systems (SRDDS) are generating a considerable amount of interest in terms of patient adherence and clinical convenience due to their controlled drug release pattern [13,14]. Injectable SRDDS can be implemented in various forms, such as non-aqueous solutions, suspensions, emulsions, liposomes, nano/microspheres, hydrogels, and so on [15,16,17]. Among them, in situ forming implants (ISFIs) can be an attractive choice for SRDDS. At the injection stage, ISFI is a relatively low-viscosity fluid that can easily pass through the injection needle [15], while after the injection, ISFI transforms into a solid state, and drug release is delayed. Thus, the easy administration and prolonged drug release at the same time are major advantages of ISFI [15,18,19]. However, this method also has limitations due to the initial burst until solidification is completed [18].

To overcome the above hurdles, we developed self-assembled microspheres by microprecipitation using PLGA, Pluronic^®^ F-127 (F127). PLGA and F127 are well known for their safety and are widely used in several FDA-approved products [20]. PLGA is one of the most commonly used polymers in biomedical fields, because of its biocompatibility, biodegradability, and controllable properties [21,22,23]. In addition, since PLGA has different physicochemical properties depending on the ratio of the hydrophobic and hydrophilic blocks, molecular weight, and modification of the terminal side chain, it is exploited in various fields such as pharmaceuticals, medical devices, and tissue engineering [24]. F127 is a triblock copolymer consisting of a PEG-PPO-PEG structure and an amphipathic material, and can be used as a surfactant. Therefore, there is the advantage that both hydrophilic and hydrophobic materials can be loaded at once [25]. In addition, the F127 solution is changed from sol to gel above the lower critical solution temperature (LCST). The LCST property could be employed as a key parameter for manufacturing ISFI [15,25,26,27,28,29,30,31,32].

In this study, we prepared DXA-loaded PLGA-F127 microspheres and investigated (1) the formulation character of DXA-loaded PLGA-F127 microspheres, (2) the implant formation behavior of the microspheres (3) and in vitro sustained-release characterization of DXA-loaded PLGA-F127 MS.

## 2. Results

### 2.1. Microprecipitation for PLGA-F127 Microspheres

**Figure 1:**
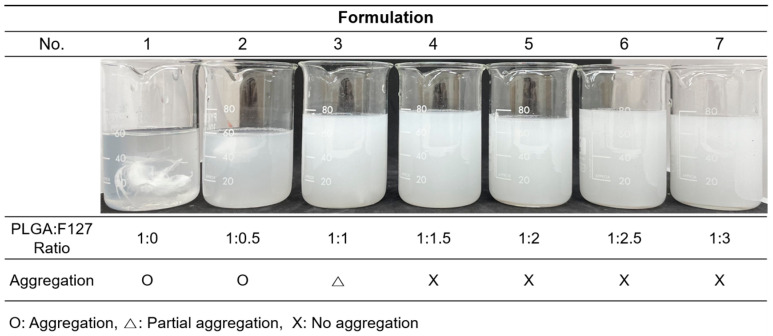
The resultants of the formulation nos. 1–7 immediately after the microprecipitation.

Figure 1 was obtained shortly after the microprecipitation of formulations nos. 1–7. (Table 1) Formulations no. 1 and 2 were particularly aggregated at the bottom of the container as soon as spraying commenced. In formulation no. 3, no huge single mass was observed, but some evident aggregations still remained; however, nos. 4–7 looked uniform and well dispersed. Formulations without or with not enough F127 could not form spherical particles. Thus nos. 1–3 were discarded.

**Figure 2:**
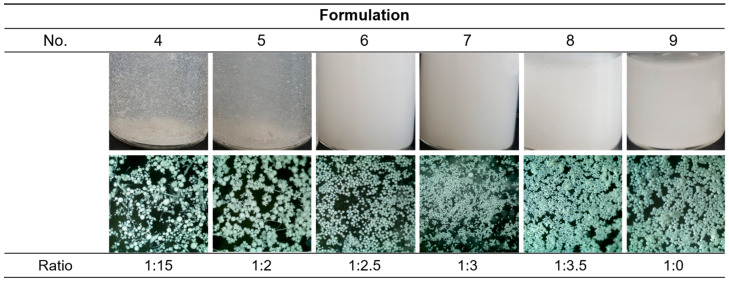
Images of the resultants after the overnight stirring. The optical microscopic images were 4× powered.

After sieving and freeze drying nos. 4–9, the lyophilates were re-dispersed in the water and observed with naked eyes and an optical microscope (Figure 2). Unlike before, nos. 4 and 5 were not dispersed uniformly and some irregular-shaped particles were observed in the microscopy. No. 4 seemed to contain more irregularity than no. 5. In nos. 6–9, all samples were dispersed well and had globular shapes, which were considered to be suitable to be injected. The formulation which was made from the higher F127 ratio seemed to contain smaller and more uniform particles. Formulation no. 9 is a PLGA-only microsphere prepared for the comparison of the presence and absence of F127. Without F127, PLGA could not form spherical particles by microprecipitation using formic acid. Thus, the conventional microemulsion method which uses dichloromethane and poly(vinyl alcohol) solution was used for formulation no. 9, which was also successfully prepared. 

**Figure 3:**
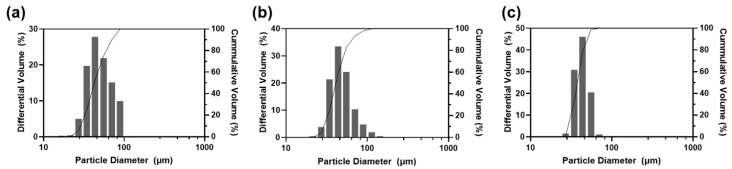
Particle size analysis against formulations (**a**) no. 6, (**b**) no. 7, and (**c**) no. 8.

For further optimization among nos. 6–8, particle size distribution was analyzed (Figure 3). All samples had diameter near to 25 μm to 100 μm; that is to say, free DXA crystals and particles that were too big to pass through the injection needle were successfully eliminated. As shown in the microscopic images (Figure 2), a higher ratio of F127 made the particles smaller and more uniform. Since this study aims to develop sustained-release drug particles, no. 6 was selected as the optimal formulation as it could form a bigger microsphere and had a smaller surface area, which are both advantageous for slow drug release.

### 2.2. Characterization of DXA-Loaded PLGA-F127 Microsperes

**Figure 4:**
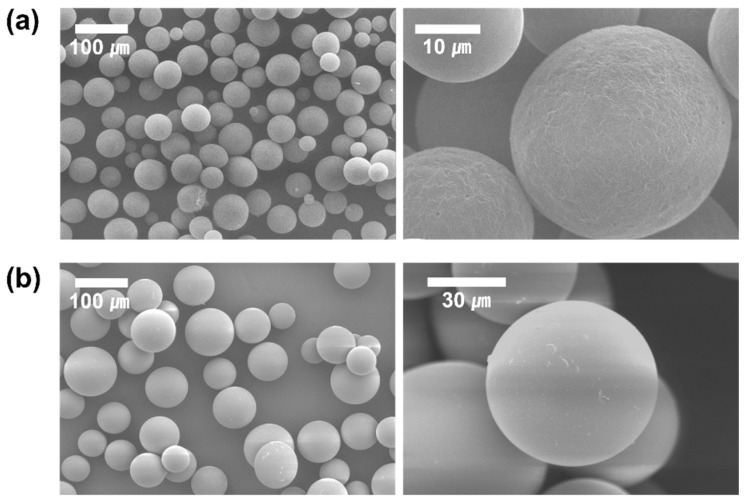
SEM images of DXA-loaded (**a**) PLGA-F127 MS and (**b**) PLGA MS.

Formulation nos. 6 (PLGA-F127 MS) and 9 (PLGA only MS) were inspected with SEM (Figure 4). As in the optical microscopy, both samples looked round and fairly uniform. Although both samples were sieved with the same sized mesh, PLGA-only MS contained a lot more larger-sized particles than PLGA-F127 MS. At the higher magnification, one difference observed was that PLGA-F127 MS had an uneven surface, but PLGA-only MS had a smooth surface.

**Figure 5:**
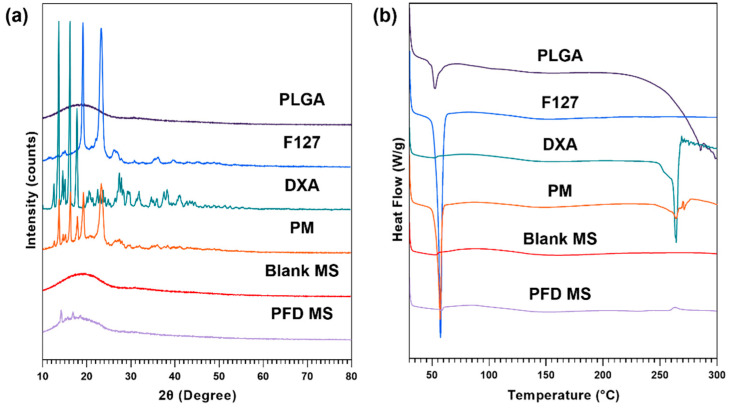
(**a**) XRD patterns and (**b**) DSC thermograms of PLGA, F127, neat dexamethasone (DXA), physical mixture (PM), Blank microsphere (Blank MS), and DXA-loaded PLGA-F127 MS (PFD MS).

To confirm the physical state and stability of dexamethasone in MS, XRD patterns and DSC curves were analyzed for DXA-loaded PLGA-F127 MS, empty MS, each ingredient, and the physical mixture of ingredients (Figure 5). In Figure 5a, dexamethasone showed characteristic diffraction peaks at 13.6°, 16.2°, and 17.8°. These were also observed in the physical mixture, but in PLGA-F127 MS, they were weakened and almost vanished. The same tendency was observed in the DSC thermogram (Figure 5b). The exothermic peak of DXA at 264.19 °C is barely recognized in the thermogram of PLGA-F127 MS, unlike in that for the physical mixture.

**Figure 6:**
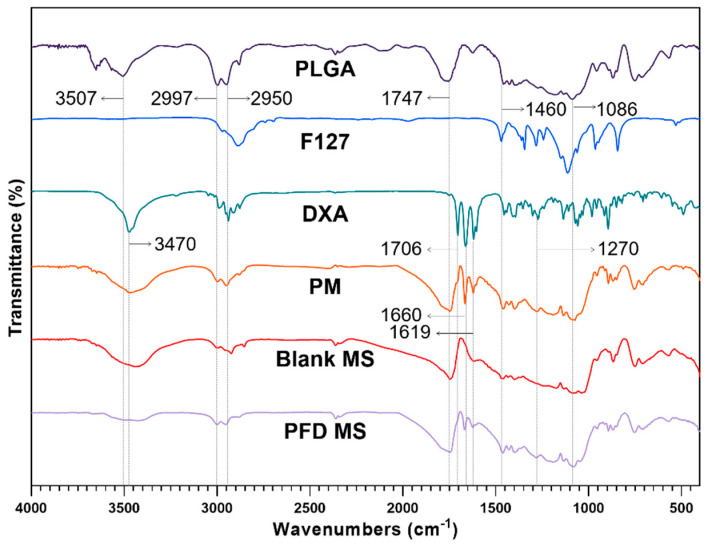
FTIR spectrums of PLGA, F127, neat dexamethasone (DXA), physical mixture (PM), Blank microsphere (Blank MS), and DXA-loaded PLGA-F127 MS (PFD MS).

The FTIR analysis was conducted to ensure that the dexamethasone was incorporated into the MS properly (Figure 6). At around 3400~3500 cm^−1^, broad stretching vibration peaks appeared due to intermolecular interaction of the hydroxyl OH group in PLGA and DXA. The typical peaks of PLGA were observed at 2997, 2950 cm^−1^ (C-H stretching), 1747 cm^−1^ (C=O stretching), and from 1460 to 1086 cm^−1^ (C-O stretching). The major peaks assigned to DXA appeared at 1706, 1660, 1619 cm^−1^ (C=O stretching), and 1270 cm^−1^ (C-F stretching). All PLGA and DXA peaks were well integrated into the PLGA-F127 MS spectrum. This indicates that DXA was successfully enclosed in the PLGA-F127 MS. 

The amount of DXA encapsulated in the MS is shown in Table 2. PLGA-F127 MS (formulation no. 6) contained 10.15% DXA, which corresponds to 50.75% of the initial amount of DXA that was input. The PLGA-only MS (formulation no. 9) contained 15.97% of DXA, corresponding to 79.85% of the initial input.

### 2.3. In Vitro Drug Release Study

**Figure 7:**
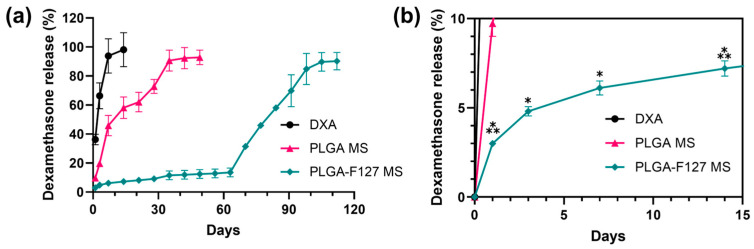
In vitro dexamethasone release profiles of neat DXA, PLGA-F127 MS, and PLGA MS. (**a**) PLGA MS shows a triphasic release profile; it also started with a slight burst (9.73%) and ended on day 35 (90.64%). However, PLGA-F127 MS released only 2.99% of DXA and this lagged phase continued to day 63 (13.50%). After that, the release rate rapidly rose and was nearly finished on day 105 (89.72%) All samples contained equivalent amount of dexamethasone and were tested in the same conditions. (**b**) The asterisks indicate significant differences compared to the previous time point.

Figure 7 exhibits the DXA release profiles of neat DXA, PLGA-only MS, and PLGA-127 MS. On day 1, 36.2% of DXA burst out and the release was almost complete on day 7 in neat DXA. PLGA MS shows a triphasic release profile; it also started with a slight burst (9.73%) and ended on day 35 (90.64%). In addition, PLGA-F127 MS released only 2.99% of DXA and this lagged phase continued to day 63 (13.50%). After that, the release rate rapidly rose and was nearly finished on day 105 (89.72%). The release profile of PLGA-F127 MS looked like a delayed biphasic pattern.

### 2.4. The Release Mechanism of PLGA-F127 Microsphere

**Figure 8:**
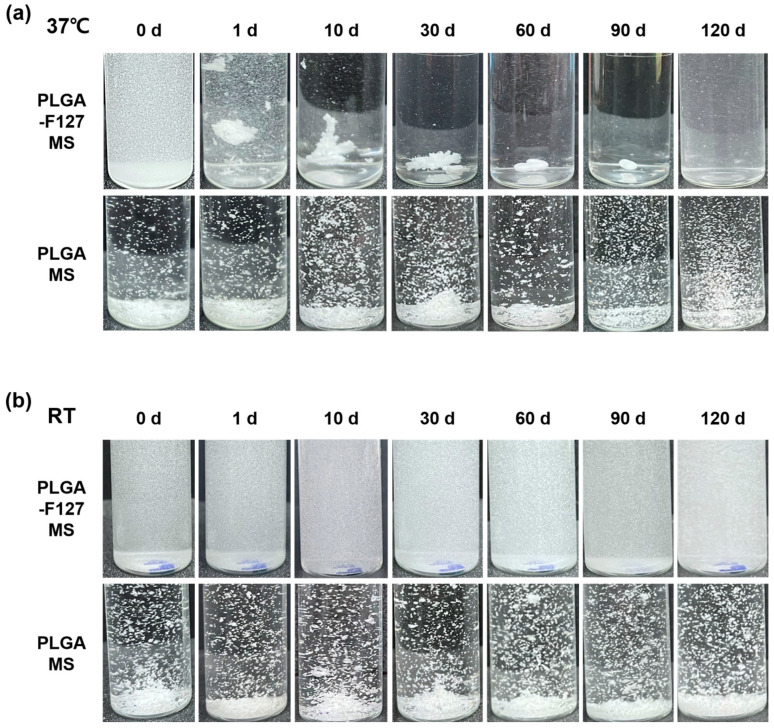
Images of PLGA-F127 and PLGA microspheres that were dispersed and incubated in water. (**a**) shows 37 °C incubated samples and (**b**) shows room-temperature samples. All samples were gently swayed with hands to float sediments before taking a picture.

To observe their self-assembly behavior, PLGA-F127 MS and PLGA MS were suspended in PBS and incubated for 90 days at 37 °C or room temperature. As shown in Figure 8a, PLGA-F127 MS started to form an agglomerate immediately after incubation began. On day 60, all of the particles were bound together and comprised a single mass; they then slowly degraded afterwards. No remaining PLGA-F127 MS was observed on day 120. However, PLGA MS did not form agglomerates and maintained a dispersed state until the observation ended. In the same experiments implemented under room temperature, neither PLGA-F127 MS or PLGA-only MS agglomerated either (Figure 8b).

## 3. Discussion

Our work revealed that PLGA-F127 microspheres loaded with hydrophobic drugs can be prepared by microprecipitation using water-miscible organic acids. Unlike the microemulsion method, the dispersed phase and continuous phase are miscible in the microprecipitation method [33]. Thus, the solvent that solubilizes the dispersed phase is diffused rapidly into the continuous phase, and solutes are extracted fast [34]. Therefore, if the solutes have a concentration that is to high, there will be a high possibility of agglomeration, like formulation nos. 1–3 in Figure 1 [34]. However, if there is enough F127, round and uniform microparticles will be formed as shown in Figure 2. These results suggest that F127 prevents hydrophobic PLGA from agglomerating and helps shape spherical particles. In this process, DXA was enclosed in PLGA-F127 MS, so the crystallinity of DXA almost vanished and became amorphous, as was proved by XRD and DSC. The FTIR results also presented successful loading of DXA in the MS without chemical transformation. However, the characteristic peaks of F127 were difficult to distinguish because almost all of the peaks overlapped with the PLGA peaks.

The diameters of the finally acquired MS were diverse and within the 25–100 μm range. Because the particles smaller than 25 μm contained a lot of free DXA, leading to an explosive initial burst, the portion of particles under 25 μm was discarded. On the other hand, the portion of particles that were larger than 100 μm could not smoothly pass through the 21-gauge needle, which is widely used in large joint injections, and so they were also excluded. Hence some of the product had to be thrown away, and so the final yield was a little bit low. But we believe that it might be improved through size optimization in future studies. The discontinuous phase of this microprecipitation method is a high viscosity solution and this can be split into particles by stirring the mixture in the continuous phase. Thus, parameters such as stirring speed, spraying speed, needle gauge used in spraying, temperature of each phase must be regulated to optimize the particle size. However, there is still another limitation related to enhancing the yield and encapsulation efficiency of this method. Because amphiphilic F127 ejects to the outer continuous phase during the spraying and the solidification, the final products have a relatively poor yield and loading capacity compared to the general microemulsion method that is commonly used to fabricate nanoparticles. Despite this imperfection, PLGA-F127 MS is still promising as an ISFI compared to conventional products. For the injectability, many ISFIs take on a gel-type form which has an inevitable initial burst, while our PLGA-F127 is both injectable and well controlled during the initial burst due to its solid microparticle form and small surface area. For producibility and enhancing yield, it may be worthwhile to explore other fabrication methods such as spray drying or microfluidics.

As out aim was to develop an intra-articular injection for a knee, the volume of the release medium was determined to be 4 mL, which is the as same as the volume of synovial fluid [35]. DXA-loaded PLGA-F127 MS took twice as long to complete the drug release than the ordinary PLGA MS; however, the drug was barely released during the initial 60 days. It is common knowledge that the ideal release pattern has a zero-order profile because the drug concentration in the blood is maintained at a constant level [36]. In terms of pharmacokinetics, the long-lagged release pattern of PLGA-F127 MS may seem undesirable. Notwithstanding this, we think that PLGA-F127 MS is still valuable as a sustained drug delivery system. This kind of release pattern is called delayed biphasic. Assuming that PLGA-F127 MS is mixed with other conventional microspheres which give 60 days of mono-or bi-phasic drug release, after the first drug release is over after 60 days, the lagged phase of PLGA-F127 will be over and second release will begin. This strategy could extend the drug release period to 4 months and may offer a nearly zero-order release profile.

**Figure 9:**
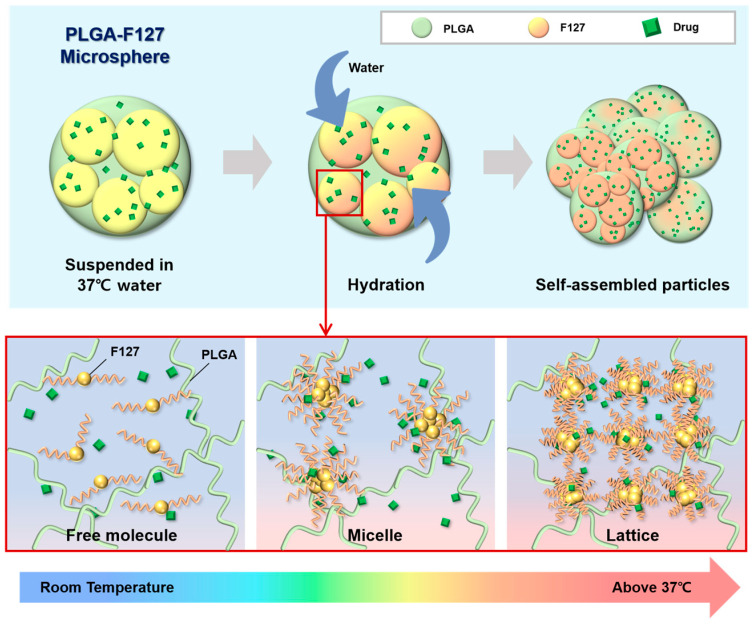
A schematic diagram of PLGA-F127 assembly.

This unusual release pattern of PLGA-F127 MS is attributed to the innate nature of F127. In Figure 8, only MS that both contained F127 and were heated were agglomerated and formed a single mass. This result implies that F127 takes on the role of helping depot formation. From this inference, we could figure out a possible model for PLGA-F127 MS’ behavior in the body (Figure 9).

When dried PLGA-F127 MS is soaked in a bodily fluid such as synovial fluid, water slowly invades the polymer matrix of the MS. At that time, amphiphilic F127 starts to solubilize whereas hydrophobic PLGA is just hydrated and swells. In the microprecipitation process, both PLGA and F127 were completely solubilized and converted into their amorphous forms and became tangled up with each other at the molecular level. Thus, entangled PLGA molecules act like a mesh which prevents solubilized F127 molecules from escaping into the water in dried microspheres. In other words, PLGA-F127 MS is like a sponge that is made of PLGA and holds a high concentration of F127 solution from a micro-perspective. As is well known, F127 could be transformed from a sol to a gel above a critical concentration and temperature [37,38]. The F127 molecules could freely change their conformation by self-assembling into a micelle because they were solubilized and their concentration was high enough in the PLGA sponge. In this circumstance, if the MS was heated, the F127 micelles could be ordered into a lattice structure. The F127 molecules located on the MS surface may lead to inter-particle packing with molecules on other MS surfaces, and then the PLGA-F127 MS can also be assembled into an enormous single mass.

**Figure 10:**
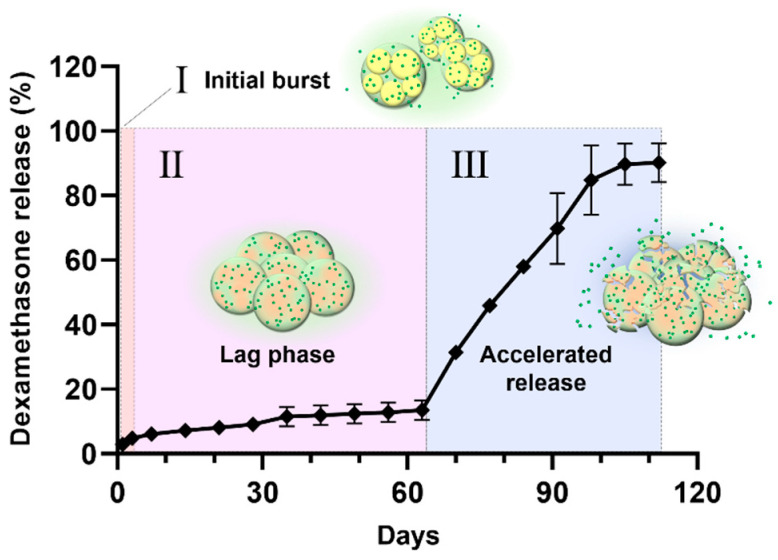
The drug release profile of PLGA-F172-dexamethasone MS across 4 months divided into 3 phases by release rate.

The drug release mechanism of PLGA-F172 MS can also be figured out by comparing the model with an in vitro drug release curve. The drug release profile was divided into three phases by release rate (Figure 10). In the first phase, including a slight initial burst, 5% DXA was released. This might be the dissolution and diffusion of DXA existing at the surface. However, this small burst ended in just 3 days because the F127 in the MS began gelation immediately after being immersed in 37 °C water. This phenomenon led to the second phase, in which the drug release was strongly restrained. As the F127 gel became harder, the polymer matrix’s density became higher, so it must have been challenging for the drug molecules to be diffused from the gelled MS [30]. Also, in the large mass formation, the total surface area of the MS got smaller and this was another factor that depressed the drug release rate [36]. Therefore, PLGA-F127 MS has an extremely long lag phase compared to ordinary MS drug carriers during the first 63 days. However, after that, the drug release suddenly accelerated until it reached 112 days. In Figure 6, the size of the implant starts to decrease from day 60 and is completely gone at day 120. Judging from the fact that the start point of degradation is correlated with drug release acceleration, it is a reasonable assumption that the erosion of the MS is the main rate-determining factor in phase 3 [36].

While recent advances in smart and effective drug delivery systems have been made in various clinical fields, the pace of development is relatively slow in musculoskeletal disease research [39,40]. In the case of musculoskeletal diseases, including osteoarthritis, patients often need to maintain their daily activities, so a delivery system that can ensure that the drug’s effects can last for a long time is clinically useful. In particular, the repeated use of glucocorticoids can cause high blood sugar levels, infections, and hormonal imbalances. Accordingly, steadily maintaining the drug’s efficacy above the therapeutic concentration for more than three months may be a good treatment option [4,6,11,24].

We are confident that our research will help to solve the difficulty in developing sustained-release drug delivery systems (SRDDS) for intra-articular DXA injection. However, to understand the comprehensive release of the drug and the final status of the microsphere, a more detailed examination of each stage, including using Nuclear magnetic resonance spectroscopy, is needed. Furthermore, there is a possibility of various applications of PLGA-F127 MS depending on its loaded drug and injection sites. However, the current study was limited by the difficulty of investigating the effectiveness of PLGA-F127 MS in vitro due to its outstandingly long drug release. Thus, future work will concentrate on carrying out in vivo studies to examine the long-term effectiveness and pharmacokinetics of PLGA-F127 MS in the arthritis animal model. During in vitro study, the biocompatibility and immunogenicity of PLGA-F127 MS should be confirmed [41].

## 4. Materials and Methods

### 4.1. Materials

Poly(DL-lactide-co-glycolide)(PLGA, 50:50, Mw = 100 kDa) was purchased from Samyoung Innovation, Corp. (Pyeongtaek, Republic of Korea). Pluronic F-127, Poly(vinyl alcohol) (Mw = 130 kDa), and formic acid were obtained from Sigma Aldrich (St. Louis, MO, USA). Dexamethasone was purchased from Bester Trading Co., Ltd. (Shanghai, China) Acetonitrile was obtained from Samchun Chemicals (Seoul, Republic of Korea).

### 4.2. Preparation of Dexamethasone-Loaded Microspheres

To establish the microsphere fabrication method, formulation nos. 1~7 were prepared. Formic acid solution containing PLGA and F127 was prepared as per Table 2. PLGA contents were fixed and F127 contents were set as multiple of PLGA contents (from 0 to 3-fold, 0.5 intervals). Dexamethasone (DXA) was 20% of the total in each formulation. To prepare the PLGA-F127 microsphere, PLGA and F127 were dissolved in formic acid, respectively. After the polymer being solubilized was completely clear, DXA was added to the F127-formic acid solution and sonicated until the mixture was homogeneous. Uniform F127-DXA solution was blended with PLGA solution, then sprayed through a 23-gauge needle and silicone tube by a pelican pump at the rate of 100 mL/min. The continuous phase was deionized water (DIW) that was stirred for 400 rpm during spraying. After that, DIW with a larger volume than the continuous phase was added and continued to be stirred overnight at 200 rpm for solvent evaporation and solidification of microspheres. Samples that succeeded in forming microspheres were sifted out with 25~100 μm sieves. The remaining solvent and free drugs were washed out by centrifuging thrice. The resultants were lyophilized. To find the optimal PLGA:F127 ratio, formulation no. 8 was prepared additionally afterwards.

For comparison, PLGA-only microsphere (formulation no. 9) was also prepared but in a different way. The reason for using the microemulsion method is that microspheres were not formed without F127 by microprecipitation using formic acid. Thus, dichloromethane (DCM) was used for the organic phase instead of formic acid and PVA solution was used for the aqueous phase to follow conventional microemulsion methods. PLGA-DXA-DCM solution was prepared and added dropwise into the 0.1% PVA solution. Afterward, followed procedures were the same as above.

The weights of lyophilized resultants were measured and yield was calculated as below.

$$Yield\;\left(\%\right)=\frac{Total\;collected\;mirosphere}{Total\;solid\;input}\times 100$$

### 4.3. Characterization of Microspheres

To confirm the appearance of particles after overnight stirring, a drop of resultant was sampled on a slide glass and observed with a Leica DM IL LED optical microscope (Leica, Wetzlar, Germany) at 10× magnification. Furthermore, the morphology of the lyophilized resultant was investigated using a SNE-4000M Mini-SEM (SEC Co., Ltd., Suwon, Republic of Korea). The samples were scattered on the carbon tape and coated using MCM-100 Ion Sputter Coater (SEC Co., Ltd., Suwon, Republic of Korea). The fixed samples were observed under 10 kV of acceleration voltage.

The size distribution of the particles was obtained using a laser diffraction particle size analyzer SALD-2300 (Shimadzu, Kyoto, Japan).

### 4.4. Drug Loading Capacity and Encapsulation Efficiency

To determine the dexamethasone loading content, high-performance liquid chromatography (HPLC) was used. We accurately weighed 5 mg of microspheres and dissolved them in 1 mL of dimethyl sulfoxide (DMSO). After the microspheres were solubilized completely, the DMSO solution was diluted with the mobile phase of HPLC 10 times, and then filtered with a 0.45 μm PTFE syringe filter. The 1260 Infinity HPLC system (Agilent Technologies, Santa Clara, CA, USA) (1260 Quat Pump VL, 1260 ALS autosampler, 1260 TCC column compartment, 1260 VWD detector) was utilized for analysis. The C18 L1 column (Capcell Pak, UG120, particle size: 5 μm, inner diameter: 4.6 mm × 150 mm) (Osaka Soda Co., Ltd., Osaka, Japan) was used. The mobile phase was acetonitrile/water 50:50 (*v*/*v*) and the flow rate was 1.0 mL/min. The sample injection volume was 10 μL. The detecting wavelength was 254 nm. All samples were prepared in triplicate and the measured contents were averaged. The calculation was made as below.

$$Drug\;loading\;\left(\%\right)=\frac{Mass\;of\;drug\;in\;microsphere}{Mass\;of\;microsphere}\times 100$$

$$Encapsulation\;efficiency\;\left(\%\right)=\frac{Actual\;drug\;loading\%}{Theoretical\;drug\;loading\%}\times 100$$

### 4.5. Fourier Transform Infrared Spectroscopy (FTIR)

The FT-IR spectra of the PLGA-F127 microsphere was studied using a Cary 600 FTIR spectrometer (Agilent Technologies, Santa Clara, CA, USA) to investigate the interaction between constituents. Microspheres and each component were ground with potassium bromide and pressed into a disc-shaped pellet. Pellets were scanned over the range of 4000 cm^−1^ to 400 cm^−1^.

### 4.6. X-ray Diffraction (XRD)

To verify the crystallinity of the dexamethasone in microspheres, 2θ was measured using an X’Pert Pro multipurpose X-ray diffractometer (Malvern Panalytical, Malvern, UK) with continuous scanning mode, in the range between 10° and 80°. The operation voltage was 40 kV and the current was 30 mA.

### 4.7. Differential Scanning Calorimetry (DSC)

The physical state of the microspheres was determined by Q100 Differential Scanning Calorimeter (TA instruments, New Castle, DE, USA). To obtain a thermogram, precisely measured samples were sealed with an aluminum pan and heated from 30 °C to 300 °C at a rate of 10 °C/min.

### 4.8. In Vitro Release of Dexamethasone

Differences between in vitro release kinetics of dexamethasone PLGA-F127 MS and PLGA MS were studied using the following method: Microspheres corresponding to 1 mg of dexamethasone were accurately weighed in the GeBaFlex-tube mini (GeBa, Yavne, Israel) and microsphere-loaded GeBaFlex tubes were soaked into an 8 mL vial which contained 5 mL of pH 7.4 phosphate-buffered saline (PBS). The vials were incubated at 37 °C in a 100 rpm shaking bath until analyzed. At the analysis points (1, 3, 7, 14, 21, 28, …, 112 days), the whole medium was replaced by fresh PBS and incubated again. As above, mediums obtained from each time point were diluted 10-fold with mobile phase and analyzed using HPLC. The amounts of dexamethasone released between each analysis point were calculated cumulatively. The experiment was conducted in triplicate and averaged.

### 4.9. Observation of Implant Formation

To observe the implant formation process of the PLGA-F127, 50 mg of PLGA-F127 MS and PLGA-only MS were placed in a 10 mL vial with 10 mL of PBS. Microsphere-containing vials were incubated in a shaking bath under the same condition as the in vitro release experiment.

### 4.10. Statistical Analysis

All numerical data had at least three replicates and were expressed as mean ± standard deviation. The statistical analysis of the results was carried out using 2-way ANOVA with Tukey’s multiple comparison test through GraphPad PRISM (version 10). Asterisks and cross symbol represent statistical significance. (*p* < 0.05 (⁎), *p* < 0.001 (⁂))

## 5. Conclusions

In this study, we provided a framework for a new sustained-release drug delivery system by successfully manufacturing a microprecipitation of dexamethasone-loaded PLGA-F127 MS. PLGA-F127 MS could only be formed under a sufficient concentration of F127 in formic acid. By XRD and DSC, the physical state and stability of the MS were confirmed. Also, using FTIR, the chemical structure of the MS was analyzed. These results evidenced that PLGA and F127 were turned into the amorphous state and adequately mixed with dexamethasone without chemical transformation. The release profile of the PLGA-MS was compared with the neat dexamethasone and PLGA-only MS. Unlike the neat drug and common PLGA MS, PLGA-MS did not show a significant initial burst; it simply retained the drug at first, and then suddenly released it after 60 days until day 105. We found that this delayed biphasic release pattern was due to the LCST nature of F127 because the release profile was accorded by self-assembly behavior of the MS. Taken together, these results suggest that our PLGA-F127 MS has the potential to be used as a novel SRDDS for local treatment. However, there are some limitations such as a low yield and encapsulation efficiency. Also, its extraordinary long-term release makes it difficult to evaluate its in vitro efficiency. To translate PLGA-F127 from the bench to the bedside, further research on producibility and in vivo efficacy will be carried out. 

## Figures and Tables

**Table 1:** Formulations of dexamethasone-loaded PLGA-F127 microspheres.

Formulation No.	Weight (mg)			Volume (mL)	
	PLGA	F127	DXA	Organic Phase	Aqueous Phase
1	100	0	25	Formic acid 1.2	DIW 25
2	100	50	37.5		
3	100	100	50		
4	100	150	62.5		
5	100	200	75		
6	100	250	87.5		
7	100	300	100		
8	100	350	112.5		
9	100	0	25	DCM 1.2	0.1% PVA 25

**Table 2:** The experimental data for the encapsulated efficiency of each formula.

Formulation No.	DXA Loading (%)	Yield (%)	Encapsulate Efficiency (%)
6	10.15 ± 0.15	35.56	50.75 ± 0.75
9	15.97 ± 0.20	56.20	79.85 ± 1.01

## Data Availability

The data that support the findings of this study are available upon reasonable request.
